# Supplementary material for: Artificial intelligence applications used in the clinical response to COVID-19: A scoping review
Source: PLOS Digit Health. 2022 Oct 17;1(10):e0000132. doi: 10.1371/journal.pdig.0000132 (PMC9931281; doi:10.1371/journal.pdig.0000132)
Supplement: Attachment — Submitted filename: Response to Reviewers_Mann.docx [file pdig.0000132.s004.docx]

Dear Dr. Pani,

Thank you for the opportunity to revise and resubmit our manuscript, “Artificial Intelligence Applications Used in the Clinical Response to COVID-19: A Scoping Review”, to PLOS Digital Health. We very much appreciate the reviewers’ thorough review and constructive comments on our manuscript.

We have revised our manuscript to address the points raised by the reviewers. We are providing two separate copies of the revised manuscript. One copy contains all revisions highlighted in track changes. The other copy is unmarked without track changes.

This document contains our responses **(bold)** to each point raised by the editor and reviewers (not bold) and highlights revisions that we have made to address these points.

We provide our responses in the following order:

- Response to comments from the editor
- Response to Reviewer 1
- Response to Reviewer 2
- Response to Reviewer 3

Please note: when we provide line number references to manuscript text, these refer to line numbers in the ‘Revised Manuscript with Track Changes_Mann’ document viewed using the ‘All Markup’ option within Microsoft Word.

Thank you in advance for your consideration.

Comments from the editor

Journal Requirements:

1. Please provide separate figure files in .tif or .eps format and remove any figures embedded in your manuscript file. Please also ensure that all files are under our size limit of 10MB.

For more information about how to convert your figure files please see our guidelines: <https://journals.plos.org/digitalhealth/s/figures>

**Response: We have removed the figures embedded in the ‘Manuscript_Mann’ file and have separately provided the following 4 figure files in .tif format. All are under the 10MB size limit.**

- **Fig1.tif**
- **Fig2.tif**
- **Fig3.tif**
- **Fig4.tif**

2. We have noticed that you have a list of Supporting Information legends in your manuscript. However, there are no corresponding files uploaded to the submission (S1 Appendix). Please upload them as separate files with the item type 'Supporting Information'.

**Response: We apologize for this oversight in our original submission. We have uploaded the supporting information file S1 Appendix.**

Additional Editor Comments (if provided):

The manuscript is interesting although there are several similar works in the field. The authors are kindly requested to provide a response, both in the revised manuscript and in a rebuttal letter, to the reviewers' criticisms, in particular those of the most critical reviewer.

**Response: In the rest of this document we respond to the points raised by all reviewers.**

**We begin by responding to the concern raised by Reviewer #1 requesting clarification on our manuscript’s added value given the existence of other review articles examining AI models that address COVID-19.**

Comments from Reviewer #1

Nice review article on AI applications developed in response to the COVID pandemic.

**Response: Thank you!**

My main concerns regard the novelty and take-home message of the article as a whole. Otherwise the article appears well-written, methodologically sound, and clear in the presentation of its results. My consideration goes more into the potential impact of a work like this (especially when its novelty is not entirely clear compared to existing similar work), especially in this phase of the COVID pandemic where priorities and surveillance activities are shifting their focus vs the initial phases of the pandemic (20-21) where most of the work reviewed in the article was performed.

Major points:

- A good number of reviews (correctly referenced in the article under evaluation) exist on this topic, e.g. Wynants et al. How is this review different? What’s its added value when a living review is already available on the same subject, especially in this phase of the pandemic? Please clarify since this is a determining factor for evaluation the original contribution of this article.

**Response: We appreciate the concern and the opportunity to clarify the added value of our review.**

**The original contribution of our review lies in its analysis of AI applications that have been deployed in clinical practice to care for patients in the COVID-19 response. Other review articles (e.g. Wynants et. al. 2020, Roberts et. al. 2021) have not attempted to identify AI applications that were deployed in clinical practice or characterize the extent of their use.**

**In order to clarify this in the manuscript, we have revised the first sentence describing the Wynants et. al. 2020 and Roberts et. al. 2021 reviews [lines 57-60, added text is italicized]: “Two separate reviews of academic literature published early in the pandemic identified hundreds of new AI models proposed to address COVID-19*, though they did not attempt to identify which models, if any, had been deployed in patient care*.”**

**We believe that our review will still be of unique value even if Wynants et. al. publish an update to their living review in the coming year, as their living review covers only academic literature, does not identify which applications have been used in clinical practice, and does not provide the same analysis as our review.**

**Our review poses, and seeks to answer, four research questions focused solely on AI applications deployed in clinical practice that are not addressed by other reviews, including Wynants et. al. These questions are [lines 71-75]:**

1. **What AI applications were used in the clinical response to COVID-19?**
2. **When, where, and at what scale were these applications used?**
3. **How do these applications relate to technology in use before the pandemic, and to what extent have applications adopted in the U.S. been reviewed by the FDA?**
4. **What evidence is publicly available to support these applications’ use in clinical care?**

**We have added text to the Objectives section of our manuscript clarifying that other reviews have not addressed these research questions [lines 69-70, added text is italicized]: “To do this, we analyzed a wide range of sources to answer the following four questions, *which have not been addressed by other reviews:”***

**Other aspects of our study that distinguish it from other reviews include:**

- **We reviewed a large number of grey literature documents to identify AI applications used in the clinical response to COVID-19 that are not described in the scientific literature. These AI applications are not covered in most other reviews. As Wynants et. al. 2020 write: “…unpublished models [not in the scientific literature] naturally fall outside the scope of this review...” (**[**https://www.bmj.com/content/369/bmj.m1328**](https://www.bmj.com/content/369/bmj.m1328)**)**
- **We review entire classes of AI applications that are not covered in Wynants et. al. 2020 or Roberts et. al. 2021. These classes of AI applications include symptom checkers and applications developed prior to the pandemic that were repurposed to care for COVID-19 patients.**
- **Our review covers academic and grey literature published from the beginning of the pandemic through the end of 2021. This covers a longer time period than Roberts et. al. 2021 which reviewed literature published through October 3, 2020 and Wynants et. al. 2020 which reviewed literature published through July 1, 2020. (note: as of Aug 29, 2022, the living review at** [**https://www.covprecise.org/**](https://www.covprecise.org/) **links to the latest published version of Wynants et. al. 2020 at** [**https://www.bmj.com/content/369/bmj.m1328**](https://www.bmj.com/content/369/bmj.m1328)**).**
- **Our review reports the documented date, extents, and locations of use for AI applications deployed in the COVID-19 response, which is not covered in other reviews.**
- **We present additional unique information not covered elsewhere, including the number and type of applications used in the COVID-19 response that 1) existed prior to the pandemic and were deployed first without modification; 2) were adapted to address COVID-19 from a pre-existing application; 3) were reviewed by the U.S. FDA; and 4) were the subject of independent evaluation studies, other types of evaluation studies, or were not the subject of any published evaluation at all.**

**As a result of these differences, there is limited overlap between the AI applications included in our review and the AI models included in other reviews. The majority of the 66 AI applications we review are not included in Wynants’ et. al. 2020, Roberts et. al. 2021, Syrowatka et. al. 2021, or other reviews.**

**To make this clear in the text, we have added the following sentence at the start of our discussion [lines 448-449]: “Most of the AI applications included in our analysis are not covered in previously published reviews.[11, 16, 18]”**

Also, apart from their deployment, number of users, and geographical coverage, I was expecting to find some sort of conclusion/discussion on the benefits (health, socio-economic, for policymaking) of the AI component of such applications. Does the well-known quote “100s of AI applications were developed for COVID… and none of them helped” still stand? Otherwise, what’s the take-home message from the review? I think this should be better elaborated and clarified in the review.

**Response: We appreciate your bringing up this important point.**

**The main take-home message from the review concerns the wide range of ways in which AI has been used in the clinical response to COVID-19 and the need for future research into their impact on patient health, given the limitations we found in currently available evidence.**

**This message is summed up in the final paragraph of our conclusion [lines 573-576]: “AI is still an emerging technology in health care, with growing but modest rates of adoption in real-world clinical care. The COVID-19 pandemic showcased the range of applications to which it is put as well as the limited evidence available on most applications that have entered clinical use.”**

**We acknowledge that our review does not provide a definitive take-home message on whether the use of AI in the COVID-19 response has provided substantial health, socioeconomic, or policymaking benefits. We state in our discussion [lines 485-488]: “As a scoping review, we did not attempt to determine the extent to which these AI applications have delivered such benefits in practice, or resulted in any unintended harms, during the pandemic. We did, however, examine the availability of evaluation studies needed to make such a determination.”**

**We engage with the well-known quote mentioned by the reviewer at the beginning of our discussion section, where we quote from and cite the MIT Technology review article [31] entitled “100s of AI tools have been built to catch Covid. None of them helped.” We write [lines 451-455]: “Our analysis supports a balanced view of AI applications’ use in addressing COVID-19. Contrary to previously published statements that no AI model has been “widely adopted”[14] or “made a real difference”[31] in the pandemic response, we found that a few AI applications were used extensively.” We continue a paragraph later [lines 458-461]: “At the same time, our analysis suggests that hopes for AI to significantly change the course of the pandemic have not been fulfilled.[32, 33] A majority of AI applications we identified were used to an unknown or limited extent. We found no evidence of a major surge in AI adoption in clinical care due to the pandemic.”**

**Our review discusses the range of proposed benefits (improving health, reducing time to care, expanding access, reducing cost) that developers and others have used to justify the use of the 66 reviewed applications. However, we found limited evidence in support of these claims — no randomized controlled trials, no studies examining access to care, just one study that measured patient health outcomes, one study that examined cost outcomes, and a few studies that examined time to care. That doesn’t mean that the reviewed AI applications have not benefited patients, but it does limit our ability to discuss the extent to which this may have occurred.**

**In order to better highlight this message, we have added a sentence at the end of our research abstract [lines 26-28]: “Due to limited evidence, it is impossible to determine the extent to which the clinical use of AI in the pandemic response has benefited patients overall.”**

– Would be worth to clarify what do you mean by “employed in the clinical setting”. Does policy-making fall into the scope? The search terms look quite generic in this regard. Later note: I think I found a better definition in inclusion criterion 1, but for the full-text screening stage. I think this should be clarified earlier and, more importantly, evaluated earlier than the full-text analysis.

**Response: We agree on the need for additional clarification of this point and have added text near the beginning of the manuscript to clearly define what we mean by “clinical” [lines 93-95]: “For the purposes of this study, we considered an application to be clinical in nature if it was used in efforts to improve patient health at the individual level as part of patient evaluation, clinical decision-making, or treatment delivery.”**

**We have also added text to inclusion criteria 1 in the methods section clarifying that applications used for public health policymaking purposes were not included [lines 153-154]: “Applications used to assist biomedical research, drug development, and public health policymaking were not included.”**

**We agree with the reviewer that the search terms are indeed quite broad and as a result yielded documents related to public health policymaking and other non-clinical areas in which AI was used in the COVID-19 response. In fact, both the search and initial screening of documents (prior to full text review) were undertaken as part of a broader project that examined AI, COVID-19, public health, and health equity topics, though this manuscript only contains the results of our study of AI in the clinical response to COVID-19.**

**We have added text at the beginning of the methods section [lines 91-92] to clarify that document search and screening were both undertaken as part of this broader project. We have also included mention of this broader project in the more detailed description of methods [see lines 109-111, 123-126].**

Minor/suggestions:

I suggest to use the PRISMA reporting guideline (especially its adaptation for scoping reviews) and associated diagram in support of the documentation efforts of the review (e.g. instead of figure 1, it would be good to adopt the PRISMA standard, which is not so different anyways)”

**Response: We appreciate this recommendation and have added a sentence to the manuscript to make clear that we followed the reporting guidelines contained in the PRISMA extension for scoping reviews [lines 83-85]: “We have followed the reporting guidelines contained in the Preferred Reporting Items for Systematic reviews and Meta-Analyses extension for Scoping Reviews (PRISMA-Scr).[24]”**

**In addition, we have reformatted Fig. 1 and added the number of duplicate records removed from search results to this figure in accordance with the PRISMA standards for flow diagrams.**

– I find figure 3 too dense and at times hard to read. I feel there’s too many different dimensions crammed into one visualization. Number of studies, color, pattern. I had to go through the legends 3 times to fully grasp all of it. Consider simplifying or redesigning”

**Response: Thank you for this comment. We agree and have removed the study type dimension (which had been represented by pattern) from the revised version of Fig. 3 to make it easier to interpret.**

Despite the review identified several applications outside the US/North American space (e.g. China and, I assume EU, in HICs) only the FDA approval was analyzed and not other equivalent quality assurance and certification procedures that are valid in places other than the US (e.g, EU medical device directive, GDPR, etc.)”

**Response: Unfortunately, we were unable to cover regulatory review of applications in China, the EU, and other jurisdictions outside of the U.S.**

**We have added a sentence at the end of the discussion section to explicitly acknowledge this limitation of our analysis [lines 545-547]: “Fifth, we limited our examination of regulatory status to applications reviewed by the U.S. FDA; a more comprehensive review of this subject would consider regulatory actions in China, the European Union, and other jurisdictions.”**

Comments from Reviewer #2

The authors carried out an extensive search for applications of artificial intelligence in the clinical response to the COVID-19 pandemic and report their findings in the article. The authors describe their search methodology in enough detail to be clearly understood. However, since it involved interviews with a limited number, albeit a diverse set, of health care stakeholders, citation chain following, and Google searches, the results are not really reproducible in detail, a fact which the authors readily admit in the "Study Limitations" section, whose inclusion is to be commended. Another strength of the article is that the Introduction clearly states the four-fold objectives of the present study. They also clearly lay out the criteria for an application to be included in their analysis as well as the template for extraction of information from these applications.

**Response: Thank you!**

Their results are presented in an organized fashion: they grouped the AI applications into 5 main categories as well as a catchall bucket. It gets somewhat tedious to read through the exhaustive enumeration of how many applications fall into further sub-divisions within each category. Tables might have been used to more succinctly present this information. An adequate number of figures is presented, although they vary in quality and readability. For example, from Figure 2, it is easy to discern date, but the thin colored lines representing each application make it difficult to tease out country.

**Response: Thank you for making this point. We agree on the need to make Fig. 2 more readable and have made several changes to improve the reader’s ability to identify which lines representing each application correspond to which location category. These changes include:**

- **We increased the colored line size**
- **We increased the jitter**
- **We widened the figure**
- **We changed the ‘Not Specified’ color from purple to gray**

Figure 4 (mislabeled as Figure 3) is very clear but could use some summary statistics to allow the main messages to jump out to the reader: e.g., x% are peer-reviewed, y% are supportive, etc.

**Response: Thank you for catching this instance of a mislabeled figure, we have now fixed the Fig. 4 label. We also agree with your suggestion and have added numbers to the Fig. 4 legend to provide summary counts of how many studies fall within each category.**

**Please note we have also made other changes to Fig. 4 in response to comments from Reviewer #1, namely simplifying it by removing one visual dimension (pattern).**

The Discussion section is refreshing to read. It summarizes the main findings and provides evidence for or against published statements that are usually accepted uncritically by most readers. For example, their findings support the notion of a "digital divide" between low-income and high-income countries, but contradict statements that AI models have not yet been used extensively, whereas in fact, some have found extensive use in the COVID-19 pandemic. They also do not find support for the often-heard claim that the pandemic has accelerated AI innovation.

They make some very important observations in this and the Conclusions section. More peer-reviewed, independent evaluations of AI applications are needed. Guidance is lacking but needed for how to conduct trials for AI applications that impact patient health and how to measure this impact. They surmise reasons for the challenges that AI applications face in gaining adoption: 1) how to show benefit beyond standard-of-care, 2) algorithm interpretability, and 3) lack of regard for clinical workflow. The authors rightly advise that clinicians should be involved in all stages of AI application development and that research should focus on implementation in real health care settings. A clear understanding of barriers and facilitators will accelerate the process of AI adoption by clinicians.

**Response: Thank you!**

Comments from Reviewer #3

This paper provides a competently done scoping review of the use of AI for largely diagnostic purposes during the start of the COVID-19 pandemic. The authors collected and reviewed a fairly large and representative sample of work. Their conclusions are based on this review and seems balanced - they note the adoption of AI for diagnostic use (clinical response) and critical evaluate claims for its efficiency (e.g. also noting possible sources of bias). Their conclusion that there is a lack of clinical trials and that more research is needed is solid and in my view an accurate assessment and necessary call.

**Response: Thank you!**
